# Advancements in Loop Cyclization Approaches for Enhanced Peptide Therapeutics for Targeting Protein–Protein Interactions

**DOI:** 10.1021/acs.joc.4c02178

**Published:** 2024-11-29

**Authors:** Lucia Lombardi, Luke A. Granger, Robin J. Shattock, Daryl R. Williams

**Affiliations:** † Department of Chemical Engineering, South Kensington Campus, Imperial College London, London SW7 2AZ, U.K.; ‡ Institute of Chemical Biology, Molecular Sciences Research Hub, Imperial College London, London W12 0BZ, U.K.; § Institute for Molecular Science and Engineering, Imperial College London, London SW7 2AZ, U.K.; ∥ Department of Infectious Disease, South Kensington Campus, Imperial College London, London SW7 2AZ, U.K.

## Abstract

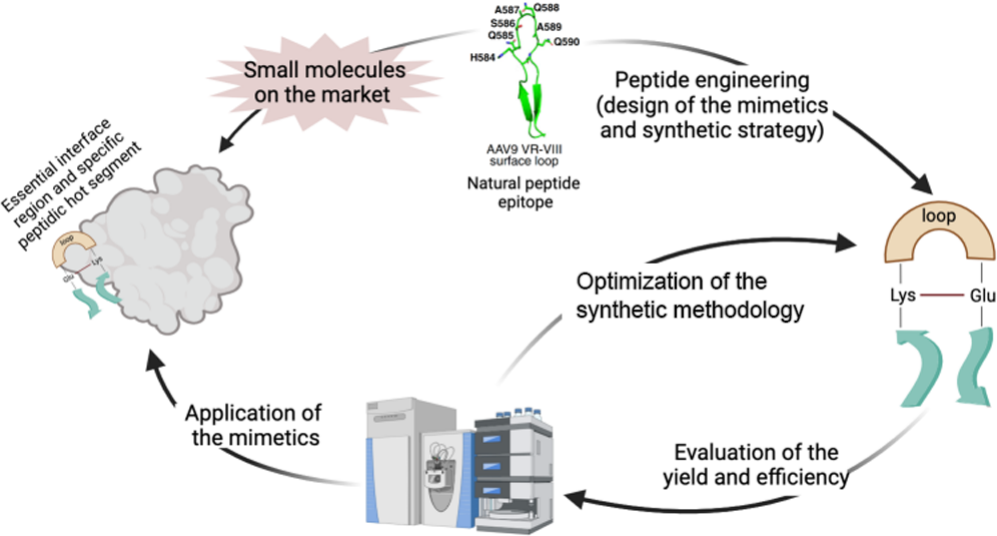

Protein–protein interactions (PPIs) are pivotal in regulating cellular functions and life processes, making them promising therapeutic targets in modern medicine. Despite their potential, developing PPI inhibitors poses significant challenges due to their large and shallow interfaces that complicate ligand binding. This study focuses on mimicking peptide loops as a strategy for PPI inhibition, utilizing synthetic peptide loops for replicating critical binding regions. This work explores turn-inducing elements and highlights the importance of proline in promoting favorable conformations for lactamization, yielding high-purity cyclic peptides. Notably, our one-pot method offers enhanced versatility and represents a robust strategy for efficient and selective macrolactamization, expanding the scope of peptide synthesis methodologies. This approach, validated through the synthesis of AAV capsid-derived loops, offers a robust platform for developing peptide-based therapeutics and highlights the potential of peptide macrocycles in overcoming PPI drug discovery challenges and advancing the development of new therapeutics.

## Introduction

Protein–protein interactions (PPIs) are essential in governing various life processes and cellular functions, influencing cell survival and death and participating in crucial biochemical reactions such as signal transduction and metabolism. Consequently, PPIs are highly significant in modern life science and medicine, being considered promising therapeutic targets for a wide range of medical conditions.^1^ However, the challenging biophysical and biochemical nature of PPIs presents obstacles to drug discovery, in both academia and industry. A significant hurdle in developing PPI inhibitors is the large, shallow, and featureless interfaces, which make it difficult for ligands to bind and pose challenges for the design and optimization of drug molecules.^2^ At PPI interfaces, loops are approximately twice as prevalent as helices or sheets.^3^ Peptide loops are structural motifs in proteins characterized by a sequence of amino acids that form a loop, often involved in critical biological functions such as binding or catalysis. Mimicking these loops can be useful in therapeutic development because they can inhibit or modulate protein interactions.^4^

There are two primary approaches to peptide loop mimicking; they are nonpeptide mimetics and peptidomimetics.^5^ While the former are small molecules designed to replicate the three-dimensional structure and function of peptide loops, peptidomimetics are peptide analogues that mimic the loop structure. Peptides and their mimetics are gradually gaining traction in various applications, notably in inhibiting diverse PPIs. Numerous examples can also be found in the treatment of diabetes, cancer, and infectious diseases. More than 65 peptide drugs have received regulatory approval, and there have been remarkable recent successes in peptide drugs for the treatment of diabetes, particularly those in the GLP-1 agonist family.^6^

Unlike helices and sheets that form uniform structures, there are no standardized models for loop mimicry due to their highly variable secondary structures.^7^ As a result, loop mimics tailored for each specific target must be identified. Constraints are often essential for the development of peptide loops.^8^ Upon incorporation of organic components, macrocycles are formed.^9^ In contrast to random-coil linear peptides, macrocycles possess a constrained ring structure, limiting their conformational variability. This constraint reduces the entropic penalty upon binding, enhancing the selectivity and affinity at the target binding site. Additionally, macrocyclic peptides may demonstrate enhanced bioactivity, lower toxicity, increased resistance to proteolysis, and improved cell-penetrating activity.^10^ The growing interest in macrocyclic peptides within the pharmaceutical industry can be attributed to their superior properties, often surpassing those of their linear counterparts. Moreover, the versatility of macrocyclic peptides allows them to be designed for virtually any drug target, thanks to the expanding toolbox for the *de novo* discovery of macrocyclic ligands.^11^ Numerous chemical approaches for cyclizing synthetic peptides have been developed, including disulfide cyclization, macrolactamization, thiol alkylation, copper-catalyzed azide–alkyne cycloaddition (CuAAC), and ring-closing metathesis (RCM).^8b^ CuAAC and RCM, while having lower cyclization yields, exhibit greater tolerance to other functional groups. Pharmaceutical companies employ different intramolecular binding strategies to make diverse macrocycles. For instance, Ra Pharmaceuticals and Polyphor focus on cycles with a single loop of amino acids. The work of Ale Therapeutics is centered on helical peptides stapled with short hydrocarbon chains. Bicyclics, peptides with two loops and a chemical connector at the core, are the main focus of Bicycle Therapeutics.^12^

Generally, the macrocyclization of peptides poses challenges because the peptide must adopt an entropically unfavorable precyclization conformation before the ends can be chemically joined. Conformational constraints in short peptides can further impede or prevent cyclization. Another obstacle is the potential for intermolecular reactions, especially at high concentrations. Conducting cyclization reactions at low or submillimolar concentrations is advisable to mitigate unwanted oligo- and polymerization reactions. On-resin macrocyclization provides an attractive alternative, offering pseudodiluted conditions and facilitating the washes of the cyclopeptide. Furthermore, in the solid phase, one end of the cycle is bound to the resin, limiting the number of conformations the peptide can adopt and increasing the probability of cycle closure.^13^

Thiol-based cyclization reactions are relatively independent of the peptide sequence, making them applicable to short peptides in which cysteines are only one or two amino acids apart.^14^ However, generally, the efficiency of cyclization reactions is strongly dependent on the chemistry, occasionally influenced by the peptide length and sequence. For instance, widely used macrolactamization reactions can also be effective, although yields may vary significantly across peptides.^8b^

This paper follows from our study on infectious diseases, specifically focusing on the development of vaccines.^1b^ Miniproteins were designed, comprising a small β-sheet, in which an essential loop connects the two strands of the sheet to establish PPIs. Our inspiration was drawn from the capsid protein of the adeno-associated virus (AAV), which, in its recombinant forms, serves as a widely utilized vehicle for *in vivo* gene replacement therapy and gene editing in both preclinical and clinical studies.^15^

**Figure 1:**
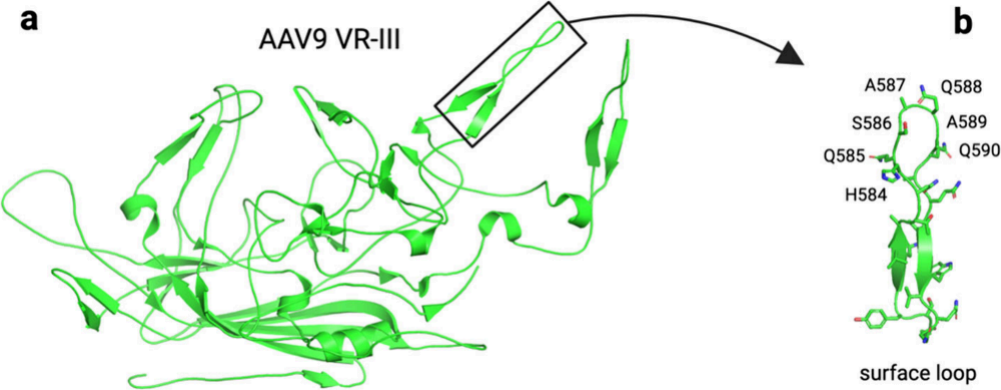
(a) VP3 displayed in cartoon format. (b) Variable region, VR-VIII, displayed in cartoon format, located on VP3. Both panels from Protein Data Bank entry 7WJW.

The AAV capsid consists of three proteins, namely, VP1–VP3, with a total of 60 monomers arranged in icosahedral symmetry in a 1:1:10 ratio. The sequence of VP3 is contained within VP2, which, in turn, is encapsulated by VP1.^16^ The monomeric structure of AAV VP3 features a highly conserved core region present across all serotypes, comprising eight antiparallel β-strands and an α-helix (Figure 1a). Additionally, loop insertions between the β-strands give rise to nine variable regions (VRs). These VRs, located on the capsid surface, are associated with specific functional roles in the AAV life cycle, including receptor binding, transduction, and antigenic specificity.^17^ This study plans to replicate the VR-VIII surface loop synthetically (Figure 1b) from adeno-associated virus serotype 9 (AAV9) and its two engineered variants identified by a direct evolution method (Table 1), as detailed by Tabebordbar et al.^18^

**Table 1:**
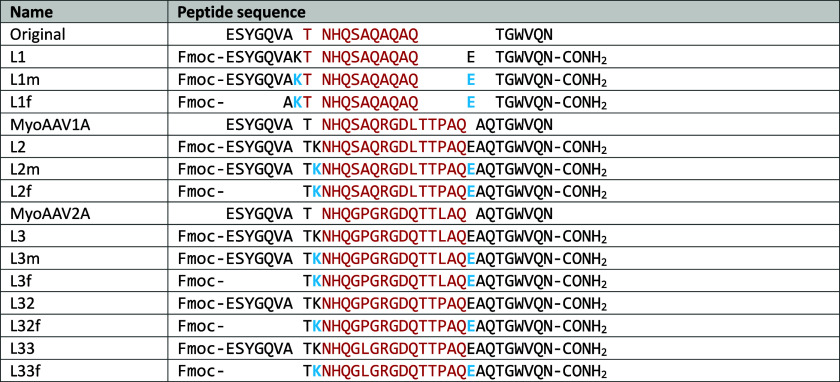
Amino Acid Sequences of the Peptides Synthesized in This Study^a^

a Residues constituting the loop region are colored red, while those forming the strands of the small β-sheet are colored black. K and E (blue) are Lys(Alloc)-COOH and Glu(OAll)-COOH, respectively.

Table 1 outlines the sequences of the original peptide and its variants, MyoAAV1A and MyoAAV2A. Macrolactamization was employed to demonstrate how this strategy can be used to cyclize peptides that typically do not undergo closure through this synthetic approach. We developed a strategy that enables the rapid and efficient synthesis of synthetic peptide loops for vaccine targeting. Our strategy, however, is a more general synthetic approach that can be applied to produce any loop sequence with any desired function.

## Results and Discussion

### Approach

The choice of the macrolactamization reaction was made due to the synthesis advantages, such as mild reaction conditions that proved to be advantageous when dealing with sensitive functional groups or substrates, chemoselectivity enabling the formation of the desired cyclic structure without unwanted side reactions, diversity of starting materials, including a wide range of amine- and carbonyl-containing compounds that provide versatility, and biological relevance as lactam rings are common in many natural products and bioactive compounds. In conclusion, these reasons make macrolactamization a valuable method for synthesizing such molecules.^13^ However, this approach also presents certain disadvantages, including steric hindrance; some substrates may not be suitable for macrolactamization due to their inherent reactivity or the absence of necessary functional groups and polymerization, which is the primary side reaction resulting from the competition between intermolecular and intramolecular reactions. As described below, these synthetic challenges were encountered and substantially resolved.

**Figure 2:**
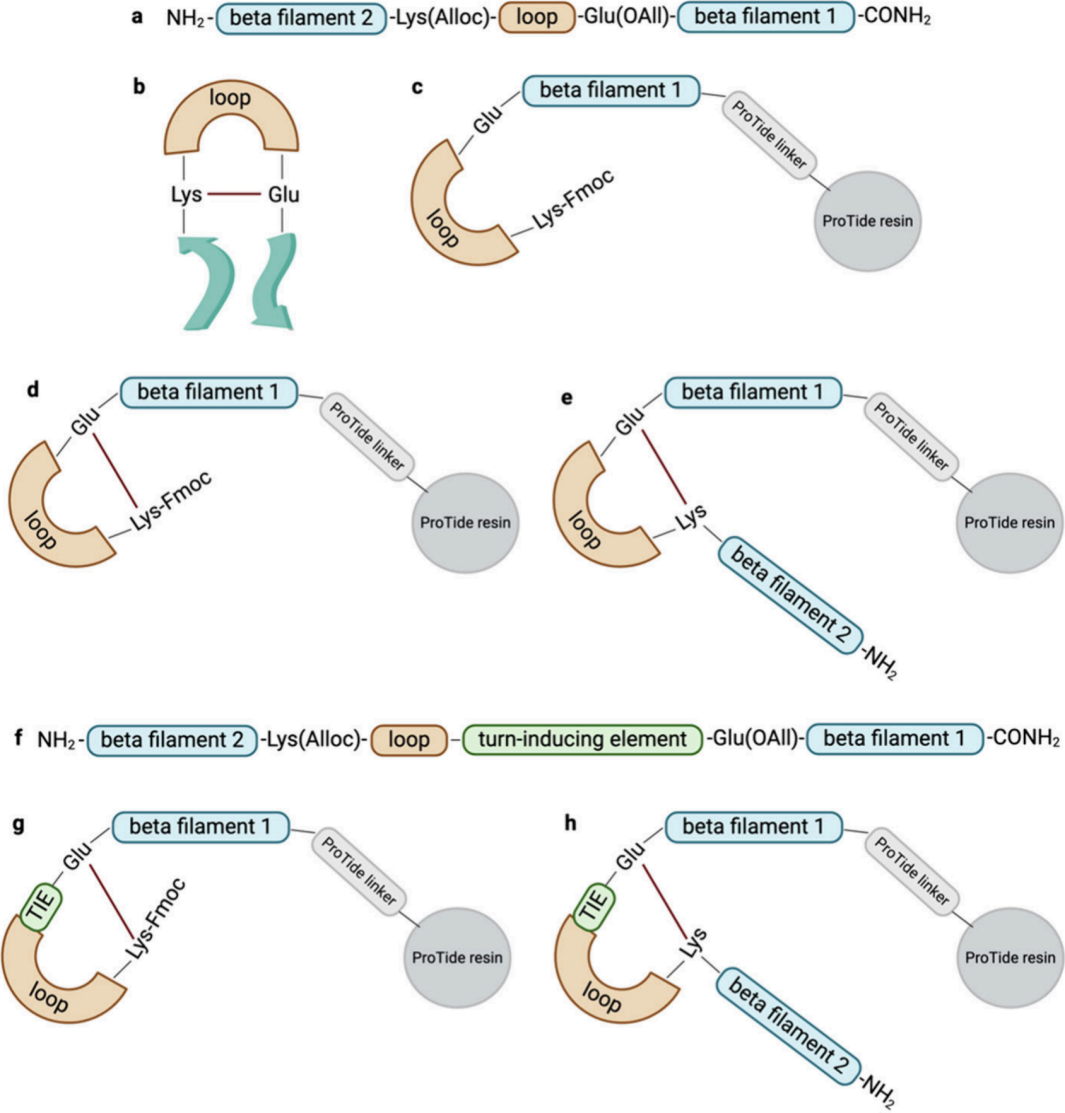
Schematic illustration describing the synthetic methodology used in this paper. Progressing from structure a to structure e in alphabetical order, the various steps of the strategy developed in this workflow are depicted. Progressing from structure f to structure h in alphabetical order, the strategy mirrors the a–e cyclization approach but incorporates the addition of the turn-inducing element (TIE).

To carry out the reaction, Fmoc-Lys(Alloc)-COOH and Fmoc-Glu(OAll)-COOH were selected and incorporated into the targeting sequences, resulting in three chimeric modifications [L1m–L3m (Table 1)]. They were precisely positioned at the beginning and end of the loop (Figure 2a) to achieve the structures shown in Figure 2b. Alloc and OAll are groups that can be selectively and simultaneously removed through a palladium-catalyzed reaction without affecting the protective groups on other residues in the peptide chain, affording L1–L3 (Table 1). Glutamic acid was chosen over aspartic acid, anticipating that the longer side chain of glutamic acid might prevent steric hindrance that could interfere with the cyclization reaction.

Work on the resin was preferred to leverage the advantages of solid-phase reactions explained above. Although the resin already provides a diluted environment for peptides, to avoid any intermolecular reactions, a resin with a low loading was used, such as 0.19 mmol/g ProTide. It is well-known that not all peptide sequences are optimal substrates for this type of cyclization (Figure 2d). However, it is demonstrated here how the addition of a turn-inducing element (Figure 2f,g) can promote the appropriate conformation that favors lactamization. Our method is general, as altering the position of Fmoc-Lys(Alloc)-COOH within the sequence allows for the generation of cyclic peptides of various sizes. Additionally, the cyclization site lacks a chiral center, eliminating the risk of epimerization.

A recently reported method employs enzymes to accelerate macrocyclization, requiring initial solid-phase peptide synthesis, cleavage, purification, enzymatic cyclization, and subsequent purification. In contrast, our one-pot method conducts all steps on the same resin, necessitating only final purification.^19^ Moreover, by using distinct protection chemistry for cyclization-involved amino acids, our method accommodates multiple lysines and glutamic acids, offering selectivity as an additional advantage. The introduction of the turn-inducing element (TIE) was also investigated, and it has been demonstrated how the presence of TIE enhances the purity and yield of the cyclic crude peptides to 96% and 98%, respectively, which are typically between 50% and 80% and between 50% and 90%, respectively, for common macrolactamization.^13^

### Discussion of the Data

**Figure 3:**
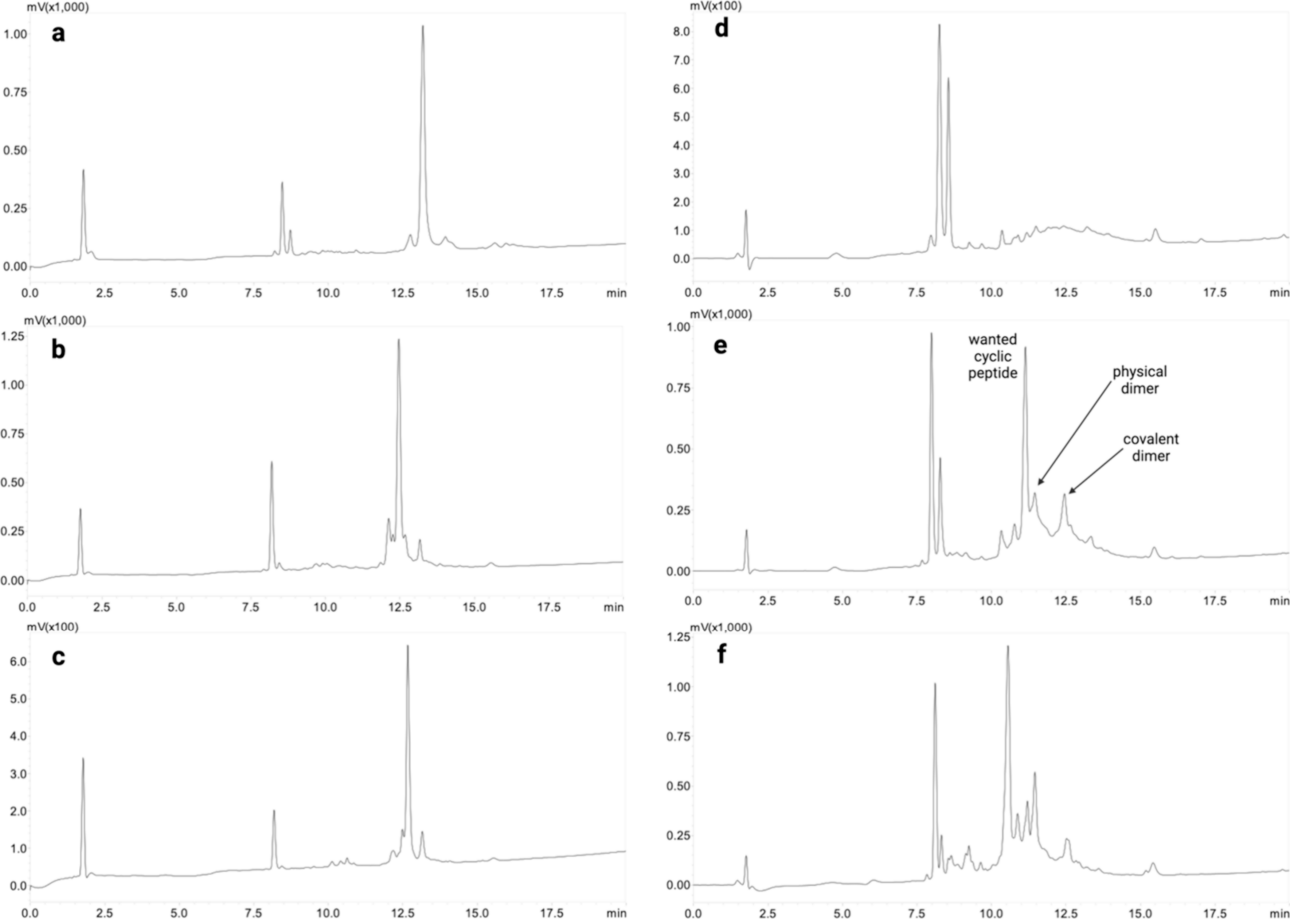
HPLC profiles of (a) linear L1f, (b) linear L2f, (c) linear L3f, (d) cyclic L1f, (e) cyclic L2f, and (f) cyclic L3f. All of the peptides were obtained on ProTide resin. The chromatograms were stopped when the wash with 95% acetonitrile commenced after an 18 min H_2_O/ACN gradient.

Peptides L1f–L3f were synthesized on a CEM Liberty Blue peptide synthesizer, ensuring the final Fmoc was on the sequence (Table 1). All three sequences were obtained on ProTide resin in high yields and purity (Table 2). In the high-performance liquid chromatography (HPLC) profiles of these linear peptides (Figure 3a–c and Figures S1 and S7), the retention time is ∼13 min. The peak observed at 8 min in all chromatograms corresponds to low-mass molecules, likely due to the resin linker fragments detaching during the cleavage process or the scavengers of the cleavage cocktail.

**Table 2:** Yields, Purities, and Mass Data^a^

name	resin	yield (%)	purity (%)		molecular weight	experimental mass (*m*/*z*)
linear L2f	ProTide	90.6	82.9		3212.45	1072.20 (M^3+^), 1607.60 (M^2+^)
linear L3f	ProTide	94.7	84.8		3168.39	1057.56 (M^3+^), 1585.79 (M^2+^)
cyclic L2f	ProTide	88.7	73.9		3071.29	1024.77 (M^3+^), 1536.78 (M^2+^)
linear L2-PEG-lipid	ProTide	98.3	96.2	after freeze-drying	3985.37	997.54 (M^4+^), 1329.50 (M^3+^)
cyclic L2-PEG-lipid	ProTide	96.9	95.4	after freeze-drying	3967.37	993.04 (M^4+^), 1323.42 (M^3+^)
linear L32-PEG-lipid	ProTide	94.2	90.3	after freeze-drying	4046.44	1013.56 (M^4+^), 1350.94 (M^3+^)
linear L33-PEG-lipid	ProTide	94.9	86.6	after freeze-drying	4062.47	1017.62 (M^4+^), 1356.16 (M^3+^)
cyclic L32-PEG-lipid	ProTide	92.7	88.6	after freeze-drying	3905.28	978.13 (M^4+^), 1303.62 (M^3+^)
cyclic L33-PEG-lipid	ProTide	92.3	80.8	after freeze-drying	3921.33	982.19 (M^4+^), 1309.04 (M^3+^)
cyclic L2m	Sieber	80.5	37.2		3806.05	953.02 (M^4+^), 1268.94 (M^3+^)

a All of the purity data were determined from the HPLC profiles of the crude peptides. The purity data of cyclic peptides were determined relative to their linear counterparts. Freeze-drying of the crude peptides (linear L2-PEG-lipid and cyclic L2-PEG-lipid) improves the purity (>95%).

Subsequently, Alloc and OAll groups were removed, and the resulting peptides, still attached to the resin, were transferred into a large bottle with 300 mL of DCM along with DIC/OxymaPure under agitation for 2 days to facilitate cyclization. Peptide L1f did not produce the cyclized form, but in its HPLC graph (Figure 3d), a short broad peak was observed, which remains unidentified. The cyclization was repeated three times with different batches of L1f, yielding the same result.

However, L2f was successfully cyclized, producing a peak at a retention time of 11 min (Figure 3e, Figures S2–S4, and Table 2).

At the retention time of 11.4 min (Figure 3e), the elution of the physical dimer of the cyclic peptide was observed. Physical aggregation is commonly encountered with cyclic compounds, especially those containing Fmoc, which is well-known for its self-aggregating properties.^20^ The peak at 12.4 min (Figure 3e) corresponds to the phenomenon of on-resin polymerization. It is evident that a small percentage of the peptide reacted with another sequence present on the resin, resulting in an intermolecular reaction that did not self-cyclize. This is not surprising considering the size of the segment forming the macrocycle (13 amino acids), which is anchored to the resin via another peptide segment of eight amino acids that represents β filament 1. Without that “peptide linker” and with a smaller macrocycle, it is expected that no intermolecular reaction would occur. However, the product of this reaction in our case is minimal. The small peaks between the retention times of 10 and 11 min, as well as that at 15.5 min, are impurities already present in the HPLC profile of the linear counterpart made on the CEM synthesizer.

With respect to L3f, no cyclization occurred, and after 4 days, the peptide remained linear (Figure 3f, 11 min). The reaction was left for up to a week but without success. After 11 min, peaks corresponding to unknown molecules were observed, which could not be identified using mass spectrometry. Other conditions, such as a temperature as high as 50 °C and the use of COMU or PyBOP as the coupling reagent, have been tested without producing good results for L1f and L3f cyclizations.

**Figure 4:**
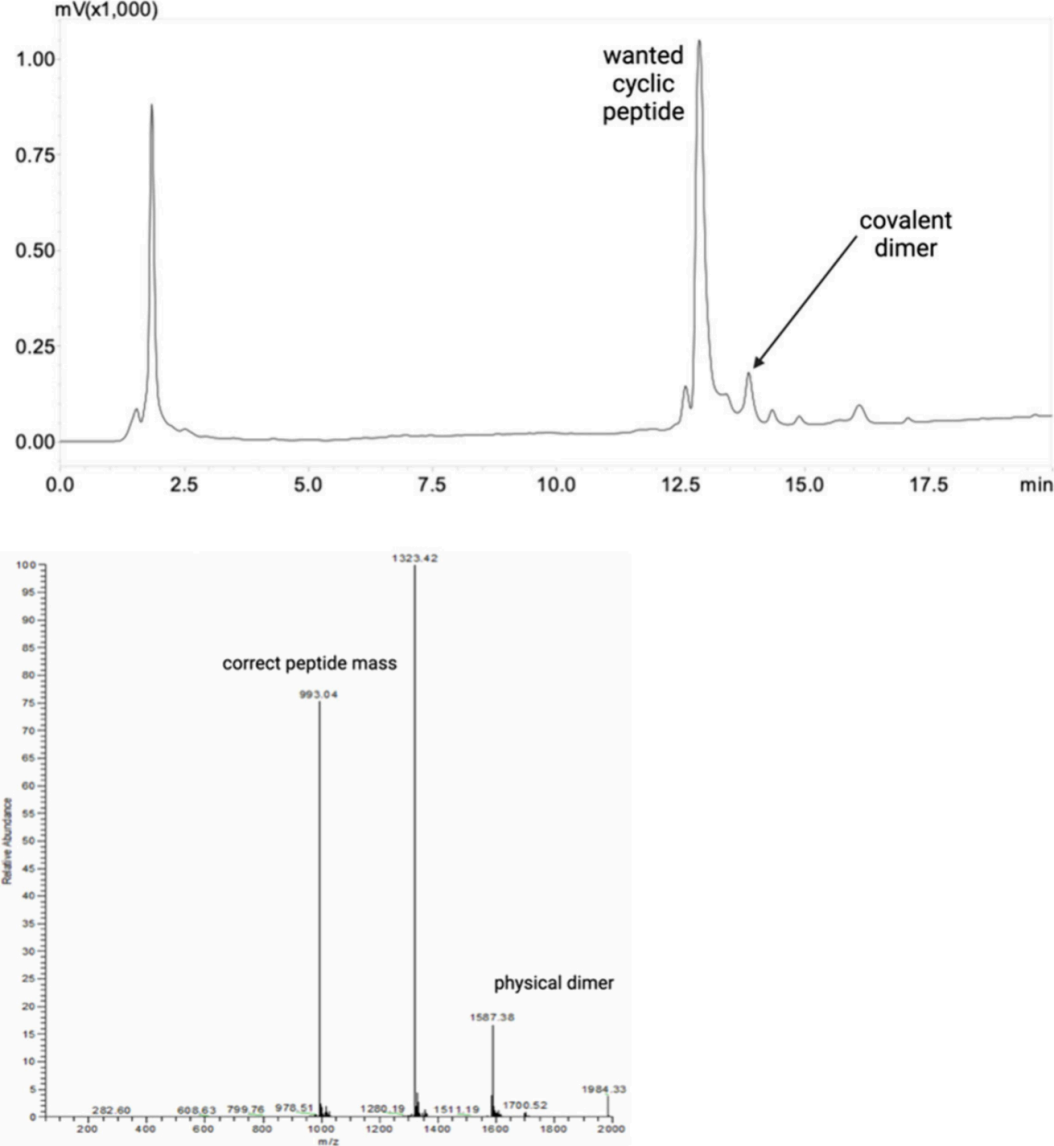
HPLC profile of the cyclic L2 peptide following the addition of a PEG spacer and lipid tails and subsequent freeze-drying. Mass spectrometry data showing the molecular mass of the monomeric cyclic L2 conjugate and the mass corresponding to the physical dimer. The chromatograms were stopped when the wash with 95% acetonitrile commenced after an 18 min H_2_O/ACN gradient. Molecular weight of 3967.37. Experimental masses of 993.04 (M^4+^) and 1323.42 (M^3+^).

Given these results, we decided to continue only with the synthesis of peptide L2f, to which the remaining amino acids were added to complete β filament 2, resulting in complete L2 (Figure 2e). The linear and cyclic versions of this peptide were obtained and completed by the addition of a PEG spacer and a lipid tail (Figure 4 and Figures S5 and S6). Figure 4 shows the cyclic L2 completed with the PEG spacer and lipid tail after freeze-drying. The peak present at 11.4 min in Figure 3b is not observed. This confirms that the peak was due to the elution of the physical dimer of the cyclic peptide that had been destroyed to give cyclic monomers with the freeze-drying process, while the peak corresponding to the covalent dimer is confirmed at 13.8 min.

To comprehend the factors that prompted the L2f sequence to cyclize while the other two did not, the types and positions of amino acids within the sequences were scrutinized. It was observed that L2 contained a proline at the end of the loop closest to the resin, while in L3f, the proline was situated in the more flexible portion of the peptide, farther from the resin. Proline is well-known for being positioned in the β turns of proteins. Due to its structure, proline possesses an essential turn-inducing capability whenever a protein needs to bend. In summary with respect to the sequences investigated in this study, proline exhibited its turning ability, allowing the peptide to adopt a semicyclic conformation and facilitating the lactamization reaction. Thus, we conclude that proline is the pivotal element around which the macrolactamization could occur.^21^

**Figure 5:**
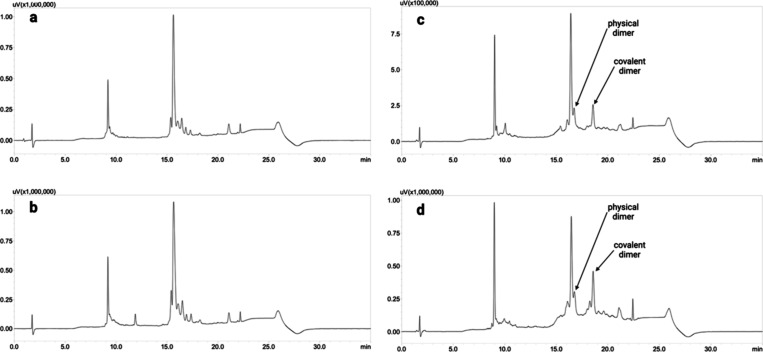
HPLC profiles of (a) linear L32-PEG-lipid, (b) linear L33-PEG-lipid, (c) cyclic L32-PEG-lipid, and (d) cyclic L33-PEG-lipid. Both peptides were obtained on a ProTide resin.

To validate this hypothesis, the L3f peptide was synthesized as two variants, L32f and L33f (Table 1). In L32f, Leu15 of the L3f peptide was replaced with Pro, while in L33f, an additional modification was made by substituting Pro7 with Leu. The aim was to assess the effect of proline at both ends of the loop. The linear (Figure 5a,b) and cyclic (Figure 5c,d) peptides (L32f and L33f, respectively) were obtained on ProTide resin, as explained above, and completed with a PEG spacer and lipid tail like the L2 peptide. Excellent results were observed, especially in the case of L32 (Figure 5c and Figure S9), for which cyclization led to a purity higher than that of L33 (Figure 5d, Figure S11, and Table 2). The higher purity (Table 2) of L32 compared to that of L33 could be attributed to the presence of an additional proline in the L32 sequence, which further promotes the precyclic structure, reducing the extent of formation of the covalent dimer that eluted at 18.3 min.

In this experiment (Figure 5d), peaks corresponding to the physical dimer and covalent dimer caused by the intermolecular reaction were observed. This is not surprising because even the L3 analogues are peptides of significant size that have undergone cyclization. The other peaks remain unknown and are not byproducts of the cyclization because they are already present in the HPLC traces for the linear counterparts.

This work demonstrated how proline could induce bending in the peptide, facilitating cyclic closure on ProTide, demonstrating the role of this amino acid as a factor to induce macrolactamization in sequences in which it might not occur spontaneously.

**Figure 6:**
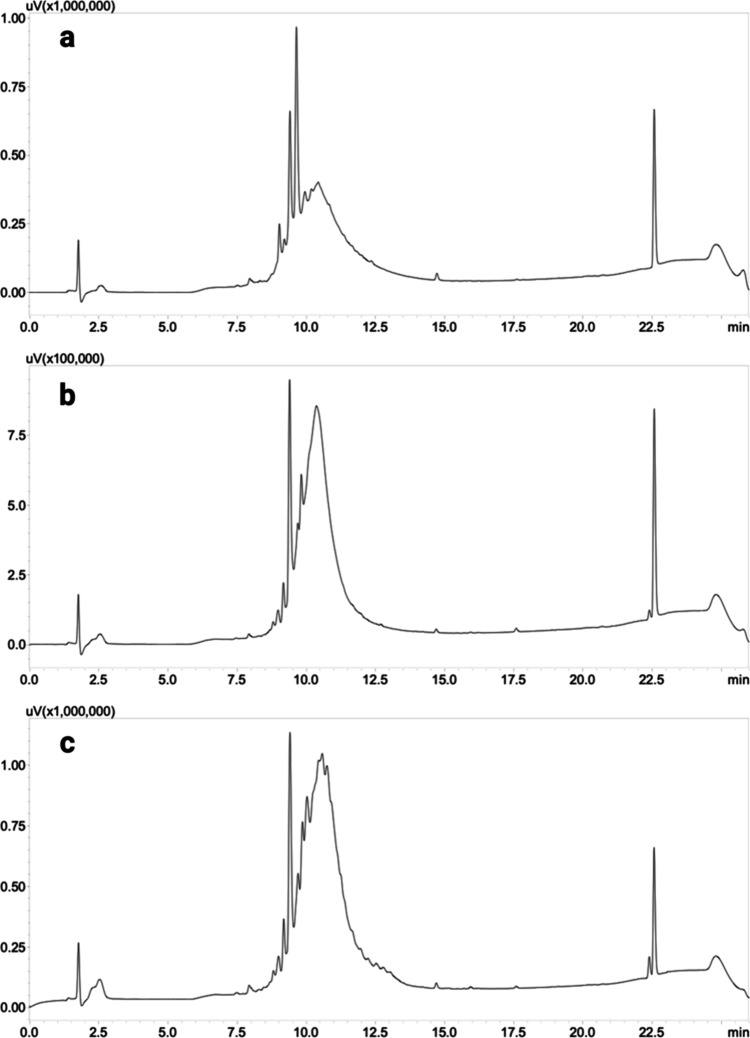
HPLC profiles of (a) cyclic L1m, (b) cyclic L2m, and (c) cyclic L3m. All of the peptides were obtained on rink amide resin.

The synthesis of peptides L1m–L3m was repeated on a different resin, specifically a rink amide MHBA resin with a loading of 0.58 mmol/g. The same results as observed with the synthesis and cyclization on ProTide resin were not obtained. Figure 6 shows the HPLC profiles of the peptides after cyclization on rink amide resin and cleavage. Broad and quite jagged peaks were observed in all three cases, including peptide L2, which yielded excellent results on ProTide resin. These peaks indicate the presence of numerous byproducts that are difficult to identify by HPLC-MS. We believe these peaks are the result of random cross-linking between two or more peptides, which occurs more frequently when the peptides are synthesized on a highly loaded resin like rink amide resin, which has a shorter linker compared to that of ProTide. The excessively high loading, ∼3 times greater than that of ProTide resin, is believed to be the reason, and resin dilution significantly affects the avoidance of side reactions for such long peptides.

**Figure 7:**
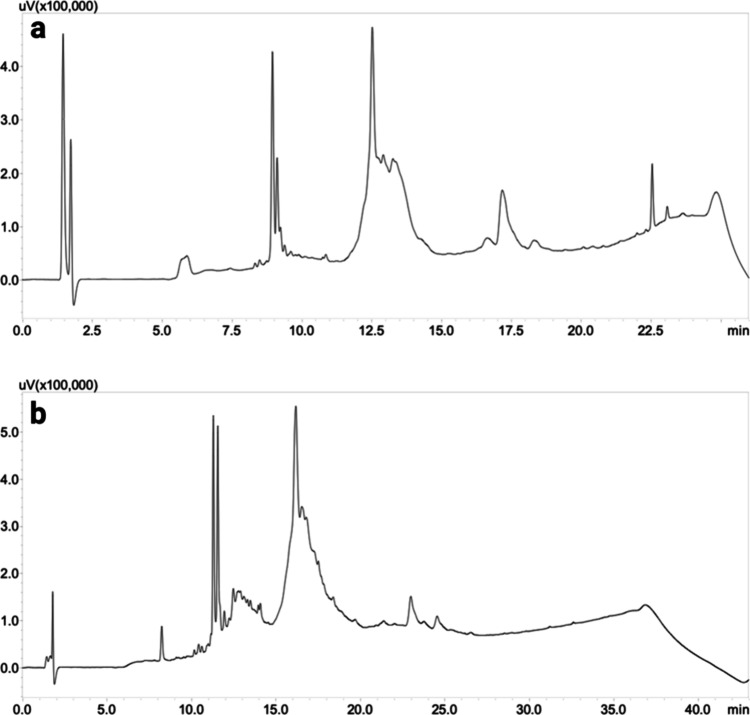
HPLC profiles of (a) cyclic L2m and (b) cyclic L2-PEG-lipid. The peptides were obtained on Sieber resin and cyclized in solution.

Subsequently, the same peptides (L1m–L3m) were synthesized on Sieber amide resin. Sieber resin, with the appropriate percentage of TFA in the cleavage cocktail, allows for the cleavage of peptides without removal of the side chain protecting groups. The aim was to determine whether the peptides could be cyclized in solution rather than on the solid phase. To this end, before cleavage from the resin, Alloc and All groups were removed using a palladium solution. After thorough washing, the peptides were cleaved from the resin while still protected. These molecules were diluted to a concentration of 0.2 mM, and cyclization was carried out in the presence of DIC/oxyma. The results confirm that the turn-inducing element, which in this case is the amino acid proline, is essential for macrocyclization in solution (Figure 7). Indeed, L2m is the only peptide that successfully cyclized, while L1m and L3m remained linear (data not shown) even in the presence of other activating agents tested, such as COMU/DIPEA or PyBOP/DIPEA.

Figure 7a shows the HPLC profile of the L2m peptide, cleaved and fully deprotected after cyclization with the protected sequence in solution. The purity of this product is lower than that of L2f cyclized on ProTide (Figure 3e and Table 2). Cyclization in solution was successfully achieved with the same molecule even after the addition of the PEG spacer and lipid tail, producing a comparable result (Figure 7b). It is deduced that additions of moieties at the N-terminus do not influence the ability to cyclize. Despite the increased number of molecular conformations, proline continues to play a predominant role, controlling the quality of the cyclization even in the case of L2m, which is a bulky molecule because of the presence of all of the protecting groups on the amino acids. For reasons not yet identified, the resin also plays an important role. The study clearly shows that the cyclic peptide peak on ProTide amide resin is purer than that obtained from the cyclization in solution.

## Conclusions

This work introduces a novel approach for designing potent PPI inhibitors using macrocyclic peptides. This approach utilizes a well-established synthetic strategy, macrolactamization, but offers new avenues for generating innovative drugs. This strategy was employed to synthesize a miniprotein containing a small β-sheet structure. A crucial loop connects the two β-strands and facilitates PPI. Our inspiration for this design stemmed from the VP3VR-VIII region of the adeno-associated virus (AAV) capsid protein.

The selection of macrolactamization was based on several advantages: mild reaction conditions suitable for sensitive functional groups, high chemoselectivity for the desired cyclic structure, diverse starting materials (amines and carbonyl-containing compounds) allowing versatility, and biological relevance as lactam rings are prevalent in natural products and bioactive molecules. However, macrolactamization also has limitations, including steric hindrance, the fact that certain substrates may not be suitable due to reactivity, and polymerization as a side reaction arising from competition between inter- and intramolecular reactions.

As discussed in this Article, these specific challenges were encountered and addressed. On-resin and in-solution cyclization methods were explored, evaluating various solid supports. On-resin cyclization not only offered the advantages of facile peptide washing and the ability to readily modify the reaction environment but also demonstrably resulted in a higher yield and a higher purity of the cyclic peptide. Additionally, we discovered that a turn-inducing element (TIE), such as proline, effectively overcomes the challenges associated with macrocyclization. This method not only unlocks a new avenue for generating highly stable and specific PPI inhibitors but also transcends the limitations of targeting large, flat interfaces. Unlike traditional small-molecule drugs, macrocyclic peptides can precisely fit the intricate contours of these interfaces, potentially leading to a new generation of drugs with unparalleled efficacy and selectivity. The simplicity and versatility of our method further pave the way for the development of a vast library of macrocyclic peptides, each with the potential to revolutionize the treatment of diseases driven by dysregulated PPIs.

## Experimental Section

### General Information

CEM ProTide resin (0.19 mmol/g, as per the supplier’s specification), Iris Fmoc-rink amide MBHA resin (0.58 mmol/g, as per the supplier’s specification), and Iris Fmoc-Sieber-PS resin (0.52 mmol/g, as per the supplier’s specification) were employed for the syntheses. Automated peptide synthesis was conducted using the CEM Liberty Blue automated microwave-assisted peptide synthesizer (CEM) in DMF as the solvent. DIC and OxymaPure served as the coupling reagents, while Fmoc deprotection was carried out using a 20% piperidine solution with the addition of 0.1 M OxymaPure. The amino acids, DIC, and OxymaPure were used at concentrations of 0.2, 0.5, and 0.5 M, respectively, with volumes of 0.5, 0.25, and 0.25 mL, respectively.

All amino acids were procured from Aapptec, and other reagents and solvents were obtained from Merck.

Analytical HPLC was performed on a Shimadzu LC20 system with Lab Solution software for data processing. The column was a Symmetry Luna C18 column (3.6 μm, 4.6 mm × 150 mm), with a flow rate of 1.0 mL/min and ultraviolet detection at 220 nm. Mobile phase A consisted of 0.1% trifluoroacetic acid (TFA) in H_2_O, and mobile phase B comprised 0.1% TFA in CH_3_CN (ACN). The applied method was a gradient from 5% to 70% B over 15 min, starting after an isocratic period of 3 min with 5% B. The pure peptide was mass-analyzed on a Velos Pro instrument (ThermoFisher Scientific), a hybrid linear trap quadrupole (LTQ)-Orbitrap mass spectrometer. The samples were directly infused into the system.

### Peptide Cyclization on Resin

The synthesis was initiated by first generating fragments of L1–L3, denoted as L1f–L3f, respectively, and modifications of L3f named L32f and L33f (Table 1), using an automatic peptide synthesizer on ProTide or L1m–L3m on rink amide resin, as previously described.^10b^ Fmoc at the N-terminus was retained.

**Scheme 1:**
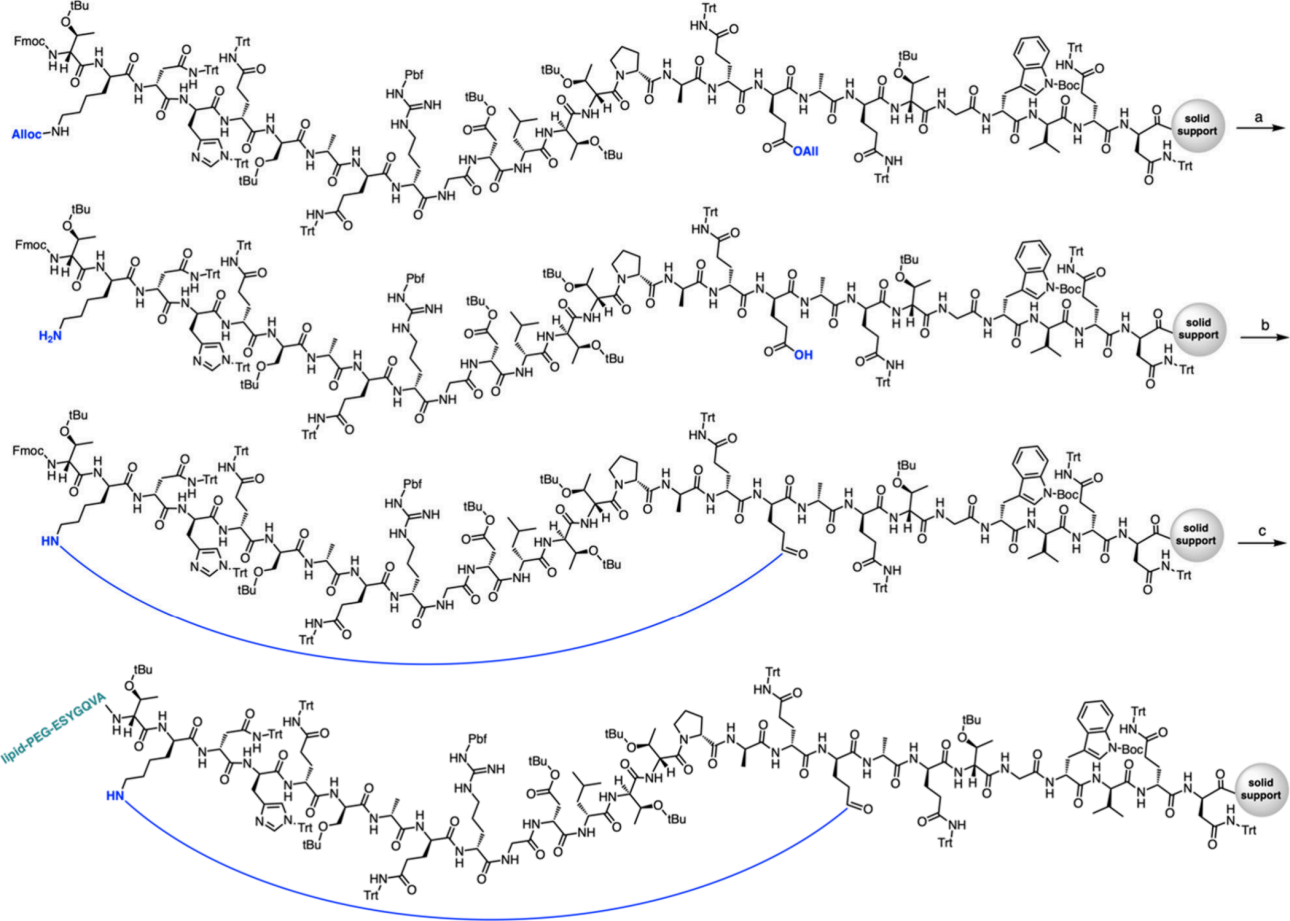
Cyclization via Macrolactamization on Resin

The resin-bound peptides were extracted from the CEM instrument for manual cyclization. Initially, treatment with 0.1 equiv of Pd(PPh_3_) and 10 equiv of PhSiH in 1 mL of DCM (equivalents with respect to the number of protecting groups to remove) was applied to remove Alloc and OAll. Subsequent washes with DCM, followed by 0.02 M sodium diethyldithiocarbamate trihydrate in 1 mL of DMF, ensured complete palladium removal. The resulting peptides were fully protected, except for one lysine and one exposed glutamic acid. After being washed with DCM, the resin was dried and transferred to a large flask containing approximately 300 mL of DCM, where lactamization was carried out (Scheme 1). The choice of a large volume of DCM aimed to prevent intermolecular interactions by minimizing collisions between peptides on different polymeric beads. Furthermore, DCM allows a better swelling of the resin than other solvents, enhancing the “dilution effect of the resin”. Cyclization was performed with 30 equiv each of DIC and OxymaPure (equivalents with respect to the peptides to cyclize) under continuous agitation. DIC was added to 300 mL of DCM without dilution, while OxymaPure was dissolved in 100 μL of DMF prior to use. The reaction progress was monitored through HPLC (after cleavage of peptides from a small portion of resin), revealing its completion after 2 days. All compounds are >95% pure as determined by HPLC before their application.

### Synthesis of the Full Peptide and Cleavage

Following the achieved cyclization, Fmoc at the N-terminus was removed, and the synthesis was manually completed with cycles of Fmoc deprotection using a 20% piperidine solution and amino acid coupling with 6 equiv of DIC and 3 equiv of OxymaPure (Figures 2e and 3c). The fully assembled peptide was detached from the resin and entirely deprotected using a 3 mL 95:2.5:2.5 (v/v) TFA/TIS/H_2_O solution for 3 h. All compounds are >95% pure as determined by HPLC before their application.

### Peptide Cyclization in Solution

The synthesis of L1m–L3m (Table 1) was initiated using an automatic peptide synthesizer on Sieber resin, as previously described.^10b^ The Sieber resin-bound peptides with Fmoc retained at the N-terminus were extracted from the CEM instrument for manual cyclization. Initially, treatment with 0.1 equiv of Pd(PPh_3_) and 10 equiv of PhSiH in 1 mL of DCM (equivalents with respect to the number of protecting groups to remove) in DCM was applied to remove Alloc and OAll protecting groups. Subsequent washes with DCM, followed by 0.02 M sodium diethyldithiocarbamate trihydrate in DMF, ensured complete palladium removal. The resulting peptides were fully protected, except for one lysine and one exposed glutamic acid.

After being washed with DCM, the peptide was cleaved from the resin with a 3 mL solution of 2% TFA and 2.5% TIS in DCM for 1 h. The peptide was precipitated in water with a volume that was 6 times greater than that used for the cleavage cocktail and then freeze-dried before use. The peptide powder, consisting of the peptide without resin but still protected on the side chains of the amino acids, was dissolved in DCM at a concentration of 0.2 mM. Cyclization was performed with 30 equiv each of DIC and OxymaPure (equivalents with respect to the peptides to cyclize) under continuous agitation. Additionally, 15 equiv of (1-cyano-2-ethoxy-2-oxoethylidenaminooxy)dimethylamino-morpholino-carbenium hexafluorophosphate (COMU) and 30 equiv of *N,N*-diisopropylethylamine (DIPEA) were tested, as were 30 equiv of benzotriazol-1-yloxytripyrrolidinophosphonium hexafluorophosphate (PyBOP) and 60 equiv of DIPEA. The reaction progress was monitored through HPLC (after full deprotection of peptides from a small solution portion), revealing its completion after 4 days.

### Yield and Purity of the Obtained Molecules

The percent yield was calculated by comparing the dry mass of the product obtained to the theoretical yield calculated with the equation
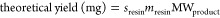
where *s*_resin_ is the resin substitution in millimoles per gram, *m*_resin_ is the resin dry mass in grams, and MW_product_ is the molecular weight of the product in milligrams per millimole. Purity is determined by comparing the peak areas of the wanted molecule to the total area.

## Data Availability

The data underlying this study are available in the published article and its Supporting Information.

## Supporting Information Available

Additional mass spectrometry data of the peptides (PDF)
