# Supplementary material for: Advancements in Loop Cyclization Approaches for Enhanced Peptide Therapeutics for Targeting Protein–Protein Interactions
Source: J Org Chem. 2024 Nov 29;90(4):1467–77. doi: 10.1021/acs.joc.4c02178 (PMC11791882; doi:10.1021/acs.joc.4c02178)
Supplement: Supplementary file 1 — jo4c02178_si_001.pdf [file jo4c02178_si_001.pdf]

Supplementary information

Advancements in loop cyclisation approaches for enhanced peptide therapeutics for targeting protein-protein interactions

Lucia Lombardi[1,2,3]*, Luke A. Granger[4], Robin J. Shattock[4], Daryl R. Williams[1]
1 Department of Chemical Engineering, South Kensington Campus, Imperial College London, London SW7 2AZ, UK
2 Institute of Chemical Biology, Molecular Sciences Research Hub, Imperial College London, London W12 0BZ, UK
3 Institute for Molecular Science and Engineering, Imperial College London, London SW7 2AZ, UK
4 Department of Infectious Disease, South Kensington Campus, Imperial College London, London SW7 2AZ, UK

*Corresponding author's e-mail address:
l.lombardi@imperial.ac.uk

Table of Contents

Figure S1. Mass spectrometry data for the linear L2f sequence........S2
Figure S2. Mass spectrometry data for the cyclic L2f sequence at 11 minutes of HPLC elution........S2
Figure S3. Mass spectrometry data for the cyclic L2f sequence at 11.4 minutes of HPLC elution........S3
Figure S4. Mass spectrometry data for the cyclic L2f sequence at 12.4 minutes of HPLC elution........S3
Figure S5. Mass spectrometry data for the linear L2-PEG-lipid sequence........S4
Figure S6. Mass spectrometry data for cyclic L2-PEG-lipid sequence........S4
Figure S7. Mass spectrometry data for linear L3f sequence........S5
Figure S8. Mass spectrometry data for linear L32-PEG-lipid sequence........S5
Figure S9. Mass spectrometry data for cyclic L32-PEG-lipid sequence........S6
Figure S10. Mass spectrometry data for linear L33-PEG-lipid sequence........S6
Figure S11. Mass spectrometry data for cyclic L33-PEG-lipid sequence........S7
Figure S12. Mass spectrometry data for cyclic L32-PEG-lipid covalent dimer sequence on resin........S7
Figure S13. Mass spectrometry data for cyclic L33-PEG-lipid covalent dimer sequence on resin........S8

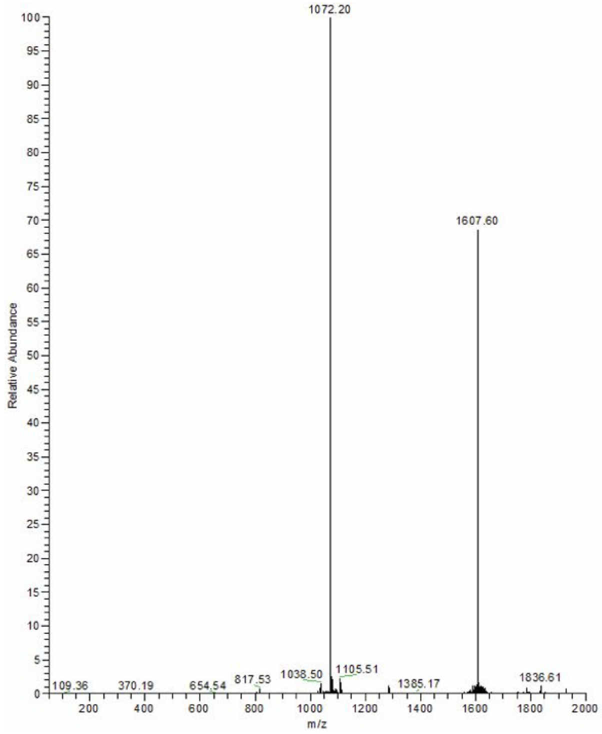

Figure S1. Mass spectrometry data for the linear L2f sequence.

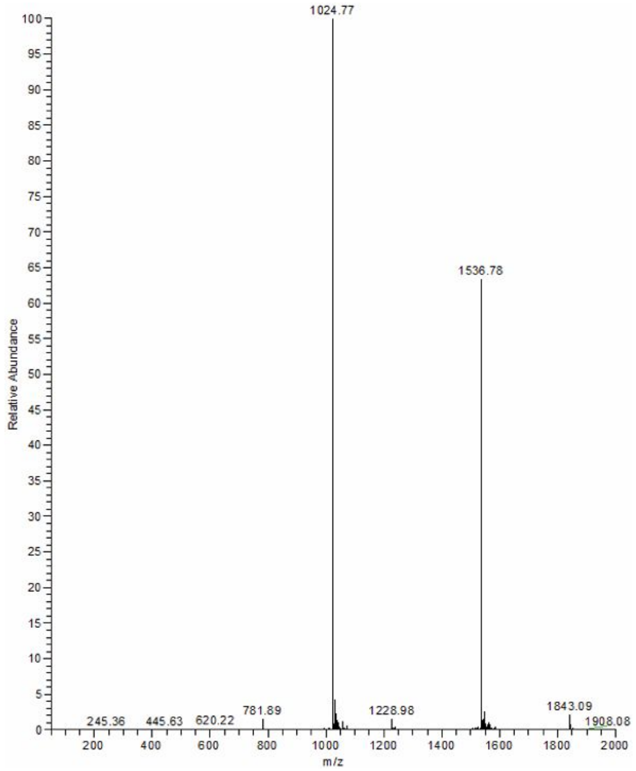

Figure S2. Mass spectrometry data for the cyclic L2f sequence at 11 minutes of HPLC elution.

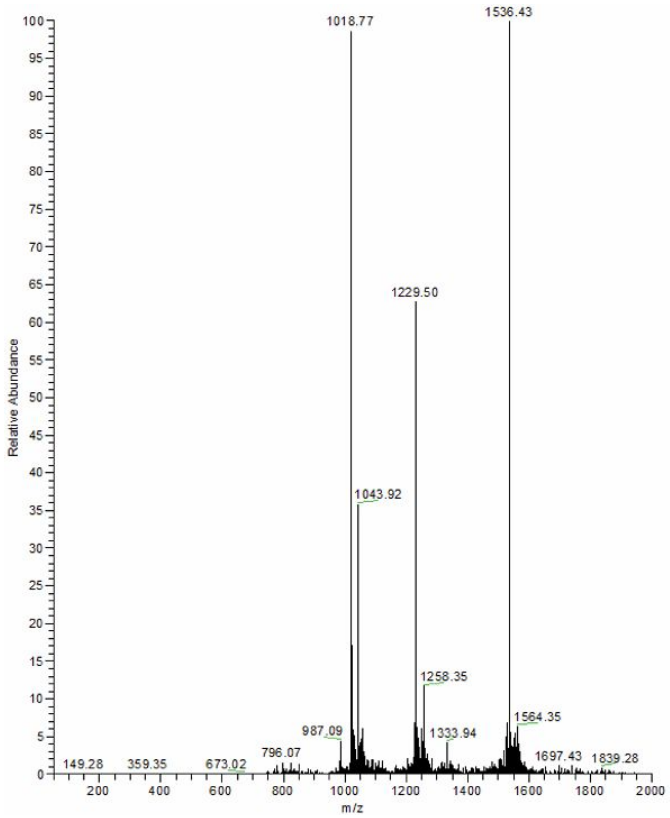

Figure S3. Mass spectrometry data for the cyclic L2f sequence at 11.4 minutes of HPLC elution.

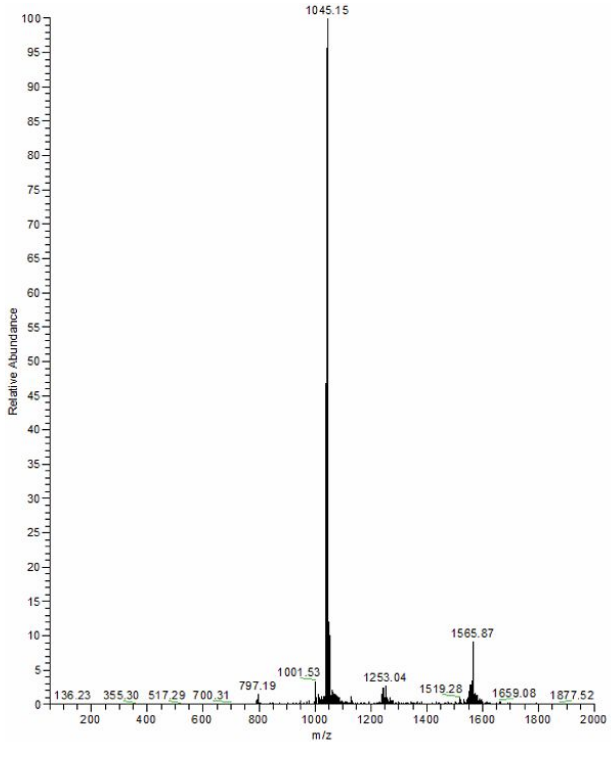

Figure S4. Mass spectrometry data for the cyclic L2f sequence at 12.4 minutes of HPLC elution.

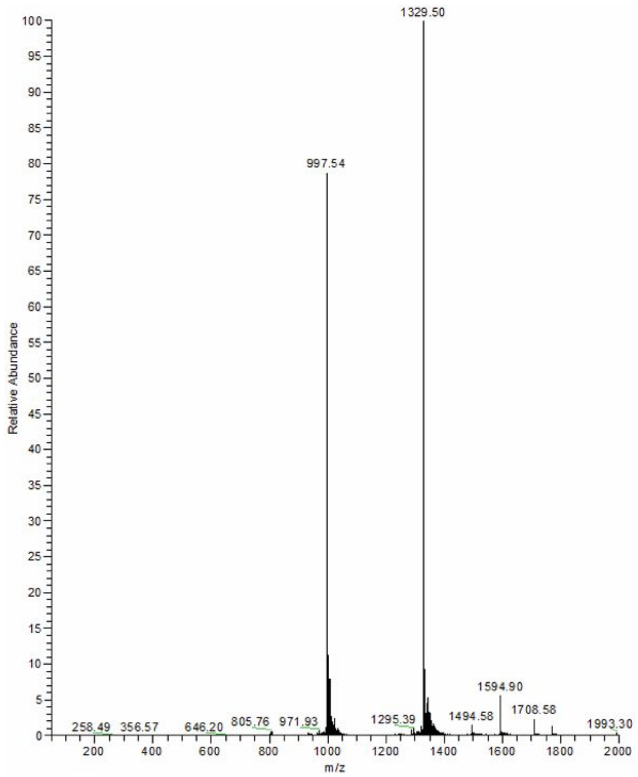

Figure S5. Mass spectrometry data for the linear L2-PEG-lipid sequence.

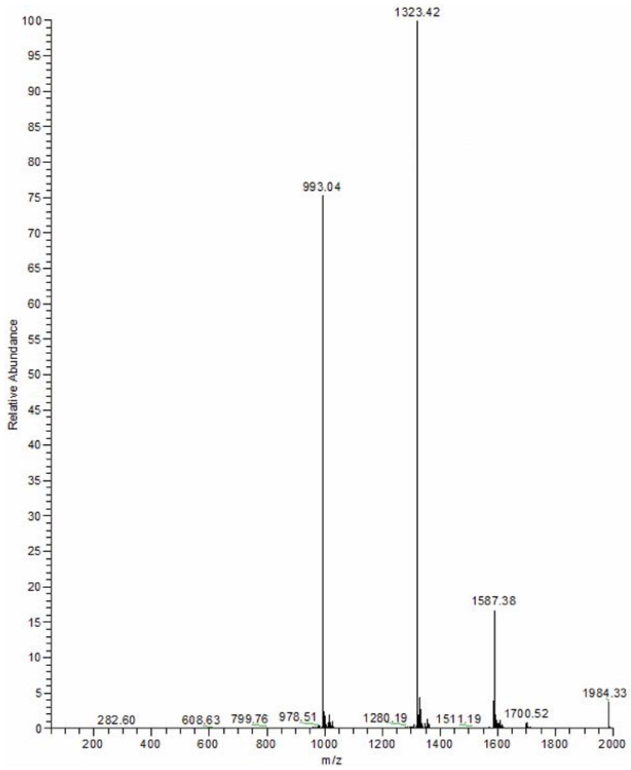

Figure S6. Mass spectrometry data for cyclic L2-PEG-lipid sequence.

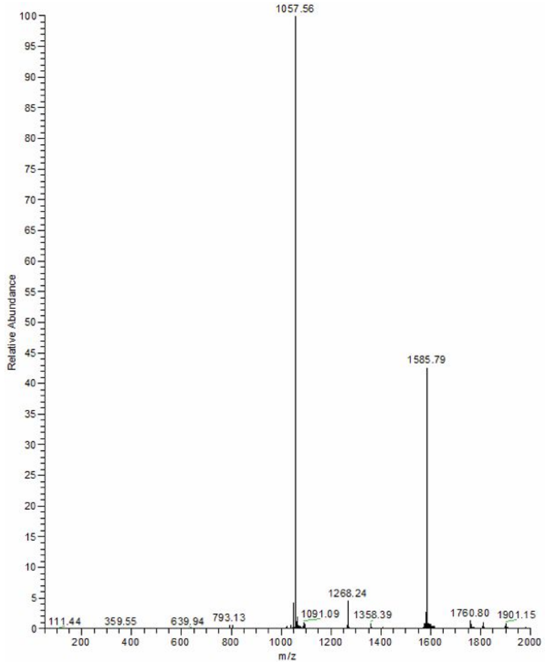

Figure S7. Mass spectrometry data for linear L3f sequence.

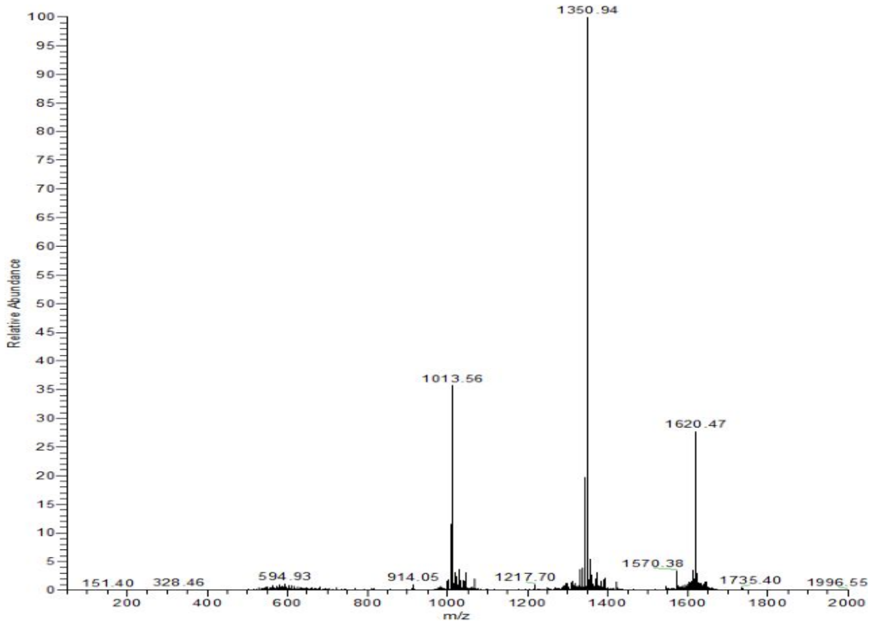

Figure S8. Mass spectrometry data for linear L32-PEG-lipid sequence.

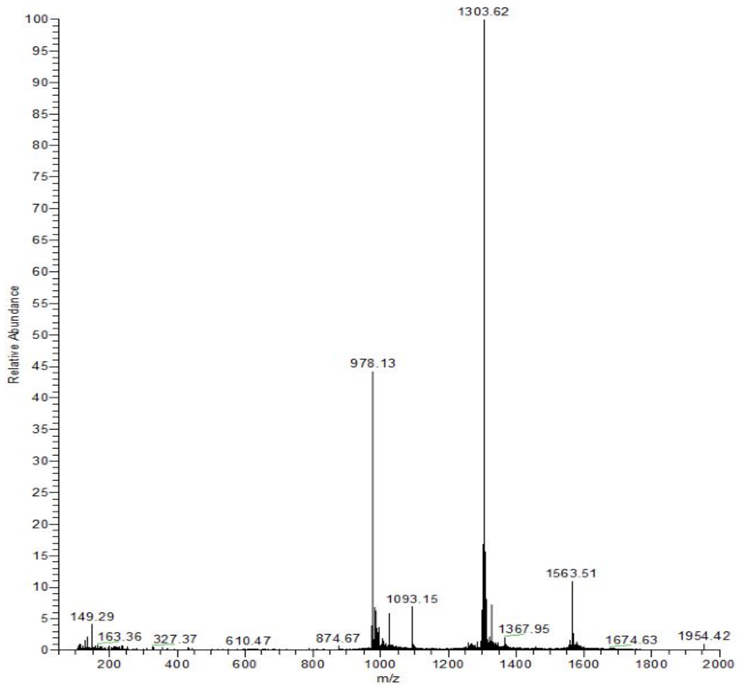

Figure S9. Mass spectrometry data for cyclic L32-PEG-lipid sequence.

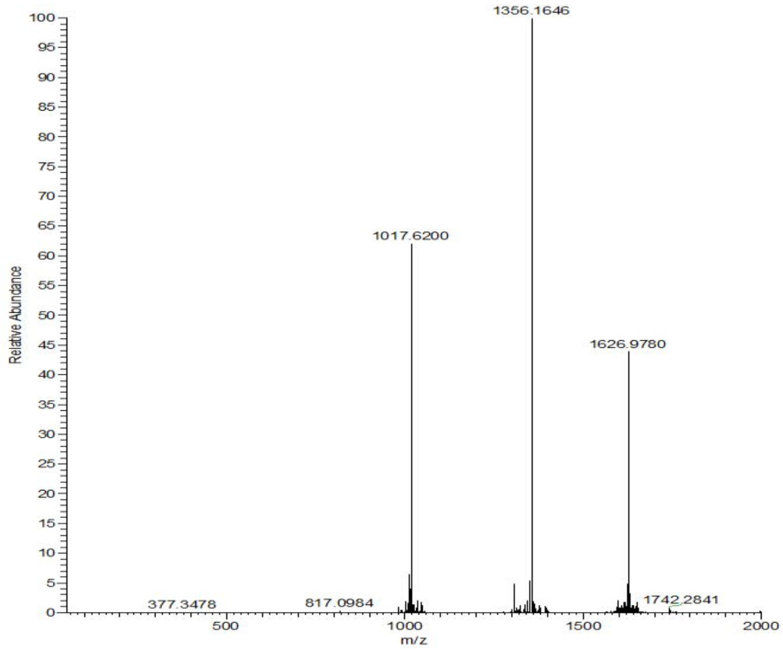

Figure S10. Mass spectrometry data for linear L33-PEG-lipid sequence.

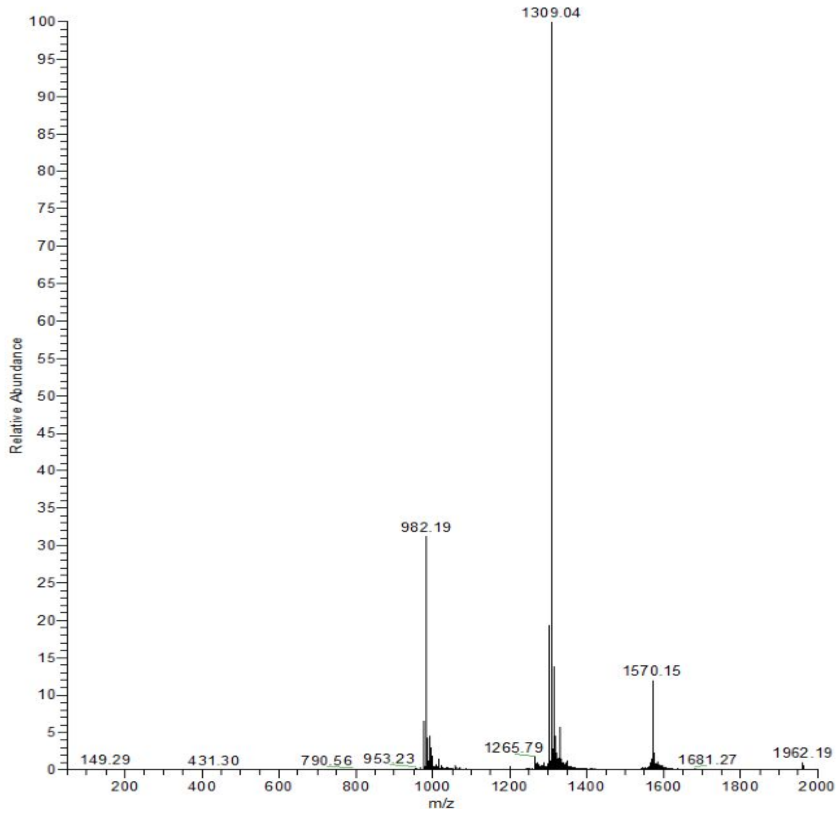

Figure S11. Mass spectrometry data for cyclic L33-PEG-lipid sequence.

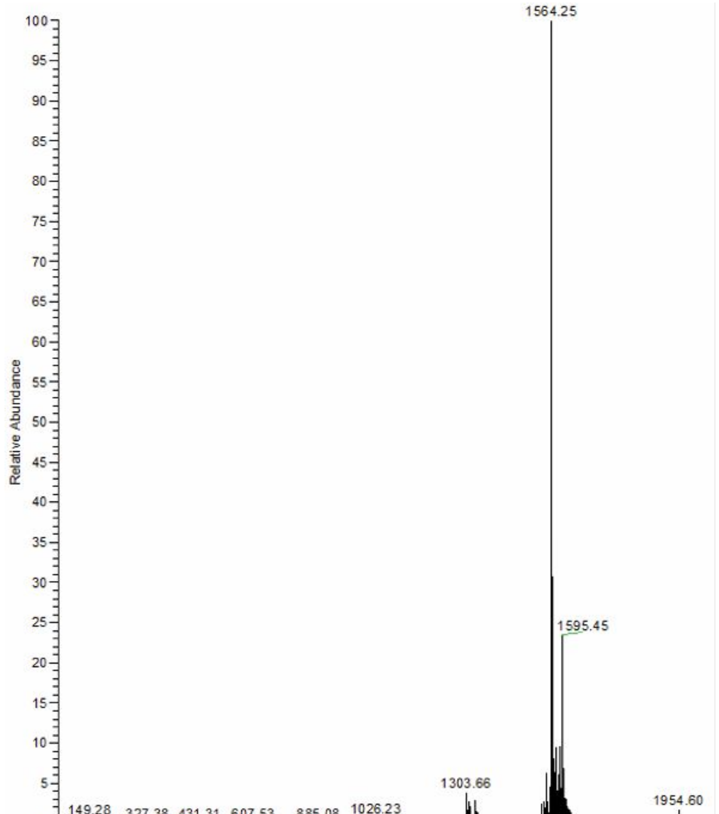

Figure S12. Mass spectrometry data for cyclic L32-PEG-lipid covalent dimer sequence on resin.

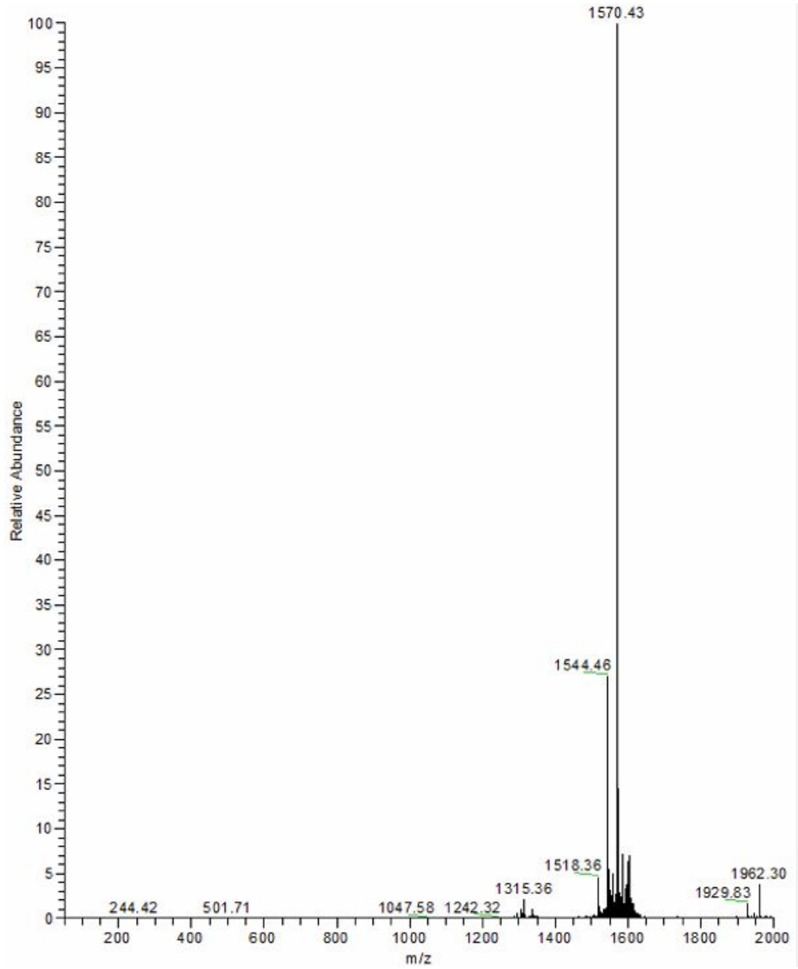

Figure S13. Mass spectrometry data for cyclic L33-PEG-lipid covalent dimer sequence on resin.
